# First report of *Sneathia sanguinegens* together with *Mycoplasma hominis* in postpartum prosthetic valve infective endocarditis: a case report

**DOI:** 10.1186/s12879-017-2654-8

**Published:** 2017-08-14

**Authors:** Iva Kotaskova, Petr Nemec, Martina Vanerkova, Barbora Malisova, Renata Tejkalova, Marek Orban, Vita Zampachova, Tomas Freiberger

**Affiliations:** 1 Molecular and Genetics Laboratory, Centre for Cardiovascular Surgery and Transplantation, Brno, Czech Republic; 2 Medical Genomics Research Group, CEITEC, Masaryk University, Brno, Czech Republic; 3 Department of Cardiosurgery, Centre for Cardiovascular Surgery and Transplantation, Brno, Czech Republic; 4 Department of Microbiology, Faculty of Medicine, Masaryk University and St. Anne’s University Hospital, Brno, Czech Republic; 5 Department of Cardiology, Centre for Cardiovascular Surgery and Transplantation, Brno, Czech Republic; 6 1st Institute of Pathological Anatomy, Faculty of Medicine, Masaryk University and St. Anne’s University Hospital, Brno, Czech Republic; 7 Department of Clinical Immunology and Allergology, Faculty of Medicine, Masaryk University, Brno, Czech Republic

**Keywords:** Infective endocarditis, Postpartum endocarditis, Sneathia, Mycoplasma, Polymicrobial infections

## Abstract

**Background:**

The presence of more than one bacterial agent is relatively rare in infective endocarditis, although more common in prosthetic cases. Molecular diagnosis from a removed heart tissue is considered a quick and effective way to diagnose fastidious or intracellular agents.

**Case presentation:**

Here we describe the case of postpartum polymicrobial prosthetic valve endocarditis in a young woman. *Sneathia sanguinegens* and *Mycoplasma hominis* were simultaneously detected from the heart valve sample using broad range *16S rRNA* polymerase chain reaction (PCR) followed by sequencing while culture remained negative. Results were confirmed by independent PCR combined with denaturing gradient gel electrophoresis. Before the final agent identification, the highly non-compliant patient left from the hospital against medical advice on empirical intravenous treatment with aminopenicillins, clavulanate and gentamicin switched to oral amoxycillin and clavulanate. Four months after surgery, no signs of inflammation were present despite new regurgitation and valve leaflet flail was detected. However, after another 5 months the patient died from sepsis and recurrent infective endocarditis of unclarified etiology.

**Conclusions:**

*Mycoplasma hominis* is a rare causative agent of infective endocarditis. To the best of our knowledge, presented case is the first report of *Sneathia sanguinegens* detected in this condition. Molecular techniques were shown to be useful even in polymicrobial infective endocarditis samples.

## Background

Although infective endocarditis (IE) is not typically of a polymicrobial nature, mixed infections have rarely been reported. They occur particularly in prosthetic IE and intravenous drug use (IDU) patients, but occasionally also in pregnancy or postpartum IE [1, 2]. *Mycoplasma hominis* has been diagnosed as an IE causative agent in up to 50% of postpartum fevers [3], which supports its potential to cause early-onset prosthetic valve IE [4]. Negative blood cultures but positive *16S rRNA* broad-range PCR in valve material and/or serology has previously been published in *M. hominis* IE [5–7]. *Sneathia sanguinegens* (formerly known as *Leptotrichia*) is a fastidious, strictly anaerobic gram-negative bacteria, part of oral cavity and urogenital tract normal microflora, and has been reported as an agent of postpartum and neonatal bacteraemia [8] and peri- or postpartum fevers [9] as well. However, to the best of our knowledge, this is the first IE case associated with this species, although IE caused by related *L. bucalis* and *L. bucalis*-like bacteria [10] has already been reported.

## Case presentation

A young woman with a history of intravenous drug use was admitted to the hospital with multiple septic pulmonary infarctions and systemic inflammatory response syndrome. Based on repeatedly positive blood cultures and echocardiogram, the diagnosis of IE was established. Urgent valve replacement was necessary using a hand-made BioMatrix one due to large vegetation destroying the tricuspid valve, including papillary muscles. During surgery, an epimyocardial electrode was inserted to avoid grade III atrio-ventricular block development. An aetiological agent was identified by blood culture as *Staphylococcus aureus*, typically associated with IDU. Isolated strain was susceptible to all tested antibiotics. The patient was treated by a combination of oxacillin 3 g every 4 h with gentamicin 80 g every 8 h for the first 10 days, continuing with oxacillin (3 g every 4 h) for another 56 days. Because of simultaneous pulmonary infection, linezolid 600 g every 12 h was added for 29 days, starting the 10th day after surgery.

One year later, the patient had a precipitate pre-term home delivery in the 34th week of gestation. Two weeks later, she was examined at an emergency ward. The patient reported having a fever and cough for 1 week prior to admission. A chest X-Ray showed no pathology, her C-reactive protein (CRP) level and white blood cell count (WBC) were 110 mg/L and 10,200 cells/μL, respectively. Outpatient therapy included oral amoxicillin with clavulanate 1 g every 8 h and paracetamol. One week later, the patient was admitted with persistent and increasing fevers, shivers, malaise, irritating cough and shortness of breath. The initial diagnosis was incipient sepsis of unknown aetiology. CRP level and WBC count were increased to 168.3 mg/L and 21,900 cells/μL, respectively. However, blood cultures remained negative. Although transoesophageal echocardiography (TEE) did not prove the relapse of IE, empiric combined antibiotic therapy with intravenous oxacillin 2 g every 4 h and gentamicin 80 mg every 8 h was initiated. Within 2 days of antibiotic treatment, CRP level and WBC count decreased to 57 mg/L and 9500 cells/μL, respectively. The patient left hospital against medical advice (AMA) with oral cefuroxime 500 mg treatment every 12 h for the next 2 weeks.

A month later, the patient was again admitted with persisting fevers of unknown origin. The patient reported health deterioration 2–3 days after the previous AMA discharge, general weakness, dizziness, shivers, and shortness of breath. Transthoracic echocardiography (TTE) and TEE reported a thick bioprosthetic valve with a rough surface, coated with vegetation prolapsing to the right atrium; the noticeable ventricular part of the valve near the septum was destroyed and massive regurgitation was observed. A Gerbode type ventricular septal defect was recorded on ultrasound. Computed tomography revealed a bilateral pulmonary embolism, while pulmonary abscesses were not present. The diagnosis of definite endocarditis was made [11] based on the presence of 1 major criterion – vegetation proved by echocardiography; and 3 minor criteria – fever, predisposing valvular conditions, and pulmonary embolism. Repeated blood cultures, urine cultures, nasal and throat swab cultures remained negative, as well as *Legionella* and *Streptococcus pneumoniae* urine antigen tests. Both short-term peripheral and central venous catheter sonicate cultures were negative. Laboratory examination showed CRP and WBC levels of 94.3 mg/L and 27,500 cells/μL, respectively. The patient responded to empirical treatment with 1.2 g intravenous amoxicillin with clavulanate every 6 h, 2 g ampicillin every 6 h, and, for the first 10 days, 200 mg gentamicin every 24 h. CRP and WBC values decreased to 29.3 mg/L and 12,600 cells/μL, respectively.

**Fig. 1:**
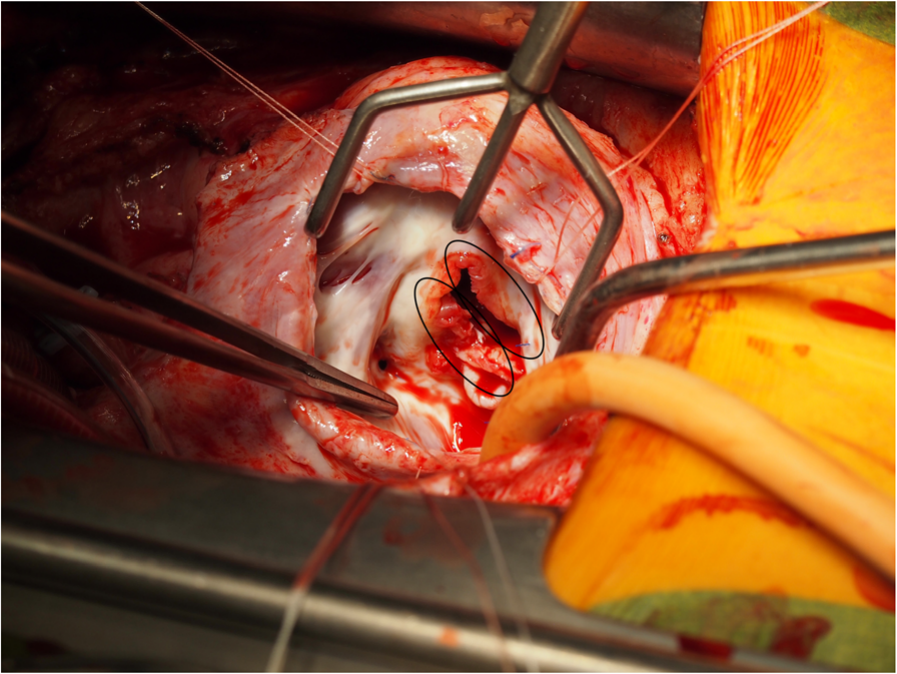
Surgery on tricuspid valve. Ellipses show inflammation affected and vegetation edged tricuspid valve

The patient underwent surgery because of a severely destroyed valve and hemodynamic failure (see Fig. 1). The bioprosthetic valve was replaced with a hand-made CorMatrix valve and sent for culture, histopathology and molecular pathogen detection. A Gerbode septal defect was closed. No immediate postoperative complication was observed.

Pacemaker implantation was necessary due to a grade III atrio-ventricular block 12 days after the surgery. In the course, the patient was afebrile, inflammatory markers decreased to 16.4 mg/L (CRP) and 11,900 (WBC per μL) and patient was discharged AMA 2 days later. By that time, the patient had been treated for 41 days with intravenous 1.2 g amoxicillin with clavulanate every 6 h and 2 g ampicillin every 6 h. Recommended following therapy for home recovery was oral 1 g amoxicillin with clavulanate every 12 h for at least the next 2 weeks.

Four months after valve replacement, no signs of systemic inflammation were present. However, TTE showed new tricuspid valve leaflet flail and III type regurgitation, the dynamics of which was recommended to be carefully monitored. Importantly, the patient’s compliance was very limited. Nine months after surgery she died from sepsis and recurrent infective endocarditis of unclarified etiology in another hospital.

### Laboratory findings

**Fig. 2:**
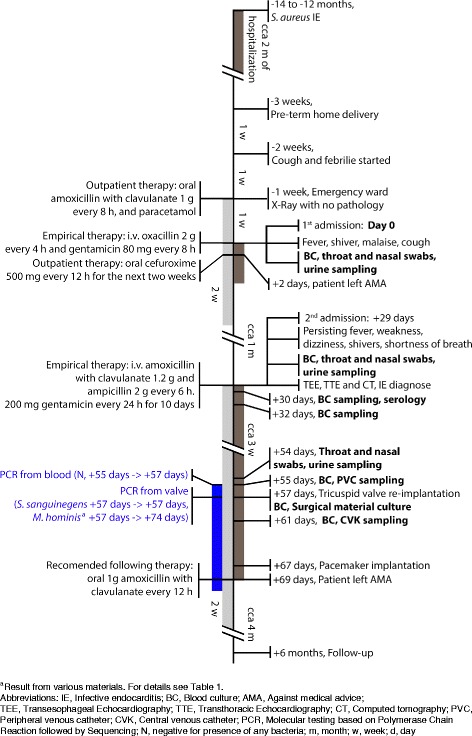
Case presentation timeline. Patient hospitalization is depicted with dark grey and prescribed antibiotic treatment with light grey rectangle. Hospital admission, symptoms, key examinations and interventions are shown. All the time points are shown in relation to the 1st admission day (day 0); + indicates days/weeks/months after and - days/weeks/months before day 0, respectively. Sampling for culture microbiological testing and serology is highlighted in bold

During the hospitalization, in total 7 repeated blood cultures were taken with no pathogen proved. All blood cultures were performed with the BacT/ALERT set (BioMérieux), in aerobic and anaerobic conditions. Results were reported after 24 h, 48 h, 6 days. For sampling details see Fig. 2. Nasal and throat swabs and urine cultures repeated three times were negative.

The replaced valve was cultured as described before [12]. Briefly, homogenized tissue was plated on sheep blood agar (Imuna), sheep blood agar with 10% sodium chloride (Imuna), and sheep blood agar with amikacin (Imuna), chocolate agar (HiMedia) and Endo agar (Imuna) for aerobic cultivation and on VL sheep blood agar (Imuna) for anaerobic cultivation. All plates were incubated at 37 °C and remained negative. Results were reported after 24 h, 48 h, and 6 days.

After that, the homogenized tissue was inoculated into a blood culture bottle and stayed negative for another 14 days. Blood from the bottle was inoculated to chocolate agar (HiMedia) and Tween agar with human blood and cultured in anaerobic conditions for another 14 days. All cultures remained negative.

Histopathology of the prosthetic valve revealed the valve’s fibrotic tissue as having dense mixed inflammatory infiltrate with an admixture of eosinophils and multinucleated macrophages, foci of necrosis with cell detritus, consistent with subacute endocarditis of the bioprosthesis. Special stainings failed to prove the presence of microorganisms.

Molecular pathogen detection results are summarized in Table 1 and Fig. 1. Broad-range molecular detection of pathogenic DNA in blood as described below was performed 1 day prior to the valve re-operation. The results were negative for the presence of any bacteria. The perioperative sample of the tricuspid valve with vegetation was examined using broad-range PCR targeting the particular bacterial *16S rRNA* region, followed by sequencing (PCR-S). DNA was extracted, amplified and sequenced as described previously [12]. Sequences were aligned with those in the GenBank, SepsiTest-BLAST [13] and 16SpathDB 2.0 [14] databases.

**Table 1:**
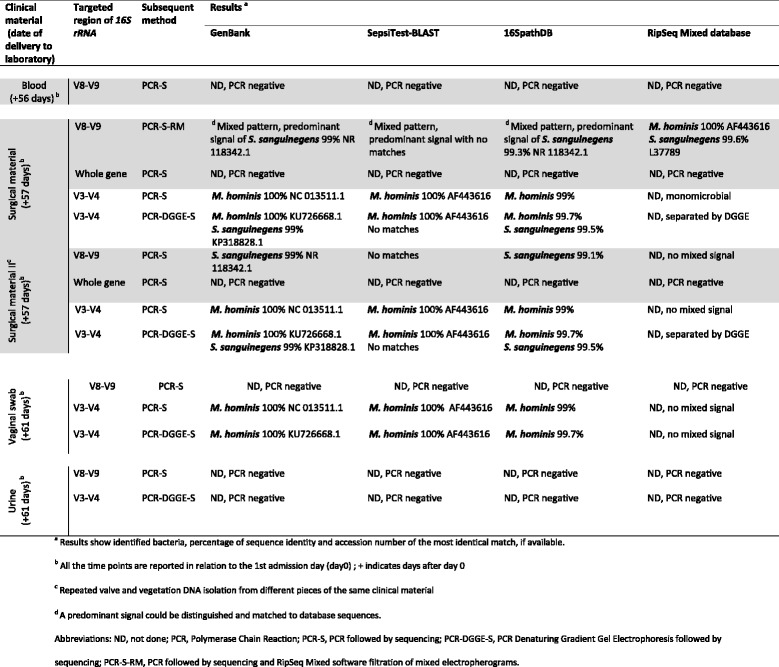
Detailed results of molecular pathogen identification

Table shows detailed results, including sample delivery dates to the laboratory. Reported results were based on routine testing of V8-V9 region and amplification of the whole *16S rRNA* gene. These results are marked with a grey background. Routine examination showed either negative results or presence of *S. sanguinegens* in tested samples. Just in one reaction, *M. hominis* was co-detected by RipSeq Mixed (V8-V9, surgical material I) and so additional, non-routine testing has been supplemented (white background). As discovered later, the false negativity for *M. hominis* was caused by incomplete annealing of common V8-V9 region and whole *16S rRNA* reverse primer

In the perioperative valve sample, sequencing the V8-V9 region of the *16S rRNA* revealed a mixed chromatogram, with predominating signal corresponding to *S. sanguinegens*. The chromatogram was further analysed by RipSeq Mixed software (Pathogenomix) as described before [15] (PCR-S-RM), showing the presence of *Mycoplasma hominis* and *Sneathia sanguinegens*. Repeated DNA isolation and amplification of the same region confirmed the presence just of *S. sanguinegens*. However, the following amplification targeting the V3-V4 region of the bacterial *16S rRNA* (forward primer: 5′- CTC CTA CGG GAG GCA GCA GT - 3′; reverse primer 5′- GGC GTG GAC TAC CAG GGT ATC TAA - 3′) was positive only for *M. hominis* in both surgical material DNA isolations. PCR results for the whole *16S rRNA* gene from both DNA isolations were negative. Based on the blood results, a urine sample and vaginal swab were requested. Urine was negative for pathogenic DNA, while coagulase-negative staphylococci and *M. hominis* were detected in the vaginal swab. Altogether, either *M. hominis* or *S. sanguinegens* or both were detected in both DNA isolations from surgical material, targeting either the V8-V9 or V3-V4 region.

To independently approve the polymicrobial nature, we performed Denaturing Gradient Gel Electrophoresis (DGGE) followed by sequencing (PCR-DGGE-S). The V3-V4 region of the *16S rRNA* was amplified (forward and reverse primer according to [16] and [17], respectively). The PCR products were separated by DGGE (6% polyacrylamide with the 30–60% denaturing gradient, electrophoresed at a voltage of 12 V for 30 min, subsequently at 120 V for 15.5 h, at 60 °C), excised out of the gel and re-amplified with the same primers lacking a GC clamp and sequenced. The results confirmed the presence of both *M. hominis* and *S. sanguinegens*. The same *M. hominis* sequence was identified in the vaginal swab (see Table 1).

## Discussion

Here we report prosthetic tricuspid valve IE with prolonged, complicated treatment and diagnosis management because of a non-compliant patient. Prosthetic heart valve and IDU history, both present in our patient, were shown as important predisposing factors increasing the risk of right-sided IE [2, 18]. In addition, a substantial increase in right-sided IE prevalence from 19 to 33.3% was demonstrated in postpartum women in meta-analysis [19], supporting the postpartum condition as a risk factor. The median time between delivery or abortion and diagnosis of IE was 3.5 weeks (24–25 days) in the same analysis. Our patient was diagnosed with definite IE 8 weeks after delivery, but incipient sepsis and possible IE relapse followed by AMA discharge from hospital 23 days after delivery, which corresponds with the reported time.

The presence of more than one bacterial agent is relatively rare in IE (4.2% of right-sided IE, [2]). Although more common in prosthetic IE and IDU patients, mixed infection is also rarely reported in pregnancy or postpartum IE [1, 2]. We do not assume contamination in our case because of the character of the detected bacteria, the negative control negativity and the confirmation of both bacteria using several independent techniques applicable for polymicrobial sample analysis, although they were not proved by culture.

To clarify *M. hominis* detection in only one surgical sample when using the V8-V9 region, but in both surgical samples when targeting the V3-V4 region, primer complementarity with reference sequences (AF443616, NR_118,342.1) was reviewed. Particularly when higher concentrations of the *M. hominis* PCR product than *S. sanguinegens* were observed using V3-V4 region and DGGE analysis, suggesting rather *M. hominis’* preferential detection*.* Indeed, a mismatch in the 3rd nucleotide from 3’end of the reverse primer (the same primer targeting V8-V9, and the whole *16S rRNA* gene) in *M. hominis*, but not in *S. sanguinegens* was found*.* This could cause preferential *S. sanguinegens* V8-V9 region amplification in repeated surgical material DNA isolation. A mismatch in complementarity with an *M. hominis* sequence and low concentration of *S. sanguinegens* could also be the cause for insufficient whole *16S rRNA* gene amplification sensitivity. Additionally, the probable higher *M. hominis* concentration in the sample could explain *S. sanguinegens* underdetection when targeting the V3-V4 region.

Repeated blood cultures remained negative throughout the whole hospitalization, as reported in up to 31% of IE ([6, 7, 20, 21]). As limiting factors of blood cultures in IE, the following were defined: 1) presence of fastidious, slow-growing or intracellular causative agents; 2) non-bacterial agent; 3) antibiotic therapy preceding blood cultures; 4) IE in patients with a permanent pacemaker [6, 7, 20–22]. In our case, two of these factors (1 and 3) were present. Molecular testing (both *16S rRNA* or taxon-specific PCR) of surgical material in blood culture negative IE cases has been repeatedly shown to have a high diagnostic value [6, 7] and can result in the lower frequency of the truly negative IE (31% of blood culture negative IE vs. 1% truly negative IE) [7].

The causal role of both pathogens is supported by the fact that both detected bacteria have been described in association with preterm delivery and amniotic fluid infections [23], as happened in our case. A similar habitat for opportunistic pathogens, preterm home delivery, and *M. hominis* proved in a vaginal swab further supports the hematogeneous dissemination of pathogens from infected amniotic fluid or genitourinary tract. Immunosuppression during pregnancy and the prosthetic nature of the valve could induce the bacterial adherence to the valve surface during bacteraemia. The presence of one microorganism may shape the environment and can prepare the niche for colonisation by another microorganism, giving rise to polymicrobial infection.

*S. sanguinegens* has been reported to be susceptible to clindamycin, amoxicillin, amoxicillin-clavulanic acid, penicillins (such as some cephalosporins of 3rd generation or some carbapenems), and resistant to vancomycin [9, 24]. *M. hominis* is an intracellular pathogen with no cell wall, susceptible to tetracyclines, some quinolones (i.e. ofloxacin) and extremely sensitive to clindamycin [25, 26]. *M. hominis* is naturally resistant to macrolides, sulfonamides, and trimethoprim in vitro [25, 26]. Both widely used antibiotic groups for IE treatment, β-lactams and aminoglycosides, are not effective for *M. hominis* treatment – no cell wall synthesis is inhibited and aminoglycosides do not cross the eukaryotic cell membrane, respectively.

The empirical antimicrobial treatment was supposedly effective for *S. sanguinegens*, detected clearly from the heart tissue sample by a routine broad-range *16S rRNA* PCR and sequencing. Indeed, the patient’s clinical conditions considerably improved. Because of the patient’s leaving the hospital against medical advice and her non-compliance, the treatment could not be modified after a final confirmation of *M. hominis* as an additional agent of IE.

## Conclusions

In conclusion, this is the first case when *S. sanguinegens* and *M. hominis,* both fastidious agents, were detected solely by *16S rRNA* broad-range PCR combined with sequencing in IE. Molecular diagnosis from a removed heart valve can be a quick and effective way to diagnose fastidious or intracellular agents and can help to set the appropriate antibiotic therapy in culture negative samples. However, all limitations such as primer complementarity of broad-range PCR have to be considered.

## Abbreviations

AMA: Against medical advice
CRP: C-reactive protein
DGGE: Denaturing gradient gel electrophoresis
IDU: Intravenous drug user
IE: Infective endocarditis
PCR: Polymerase chain reaction
PCR-DGGE-S: Polymerase chain reaction – denaturing gradient gel electrophoresis – sequencing
PCR-S: Polymerase chain reaction – sequencing
PCR-S-RM: Polymerase chain reaction – sequencing – RipSeq Mixed
TEE: Transoesophageal echocardiography
TTE: Transthoracic echocardiography
WBC: White blood cell count
